# Patients direct costs to undergo TB diagnosis

**DOI:** 10.1186/s40249-016-0117-x

**Published:** 2016-03-24

**Authors:** Rachel M. Anderson de Cuevas, Lovett Lawson, Najla Al-Sonboli, Nasher Al-Aghbari, Isabel Arbide, Jeevan B. Sherchand, Emenyonu E. Nnamdi, Abraham Aseffa, Mohammed A. Yassin, Saddiq T. Abdurrahman, Joshua Obasanya, Oladimeji Olanrewaju, Daniel Datiko, Sally J. Theobald, Andrew Ramsay, S. Bertel Squire, Luis E. Cuevas

**Affiliations:** Liverpool School of Tropical Medicine, Pembroke Place, Liverpool, L3 5QA UK; Zankli Medical Center, Abuja, Nigeria; Bingham University, Nassarawa State Abuja, Nigeria; Medical Faculty, Sana’a University, Sana’a, Yemen; National Tuberculosis Institute, Sana’a, Yemen; Bushullo Major Health Centre, Awassa, Ethiopia; Tribhuvan University Institute of Medicine, Kathmandu, Nepal; Armauer Hansen Research Institute, Addis Ababa, Ethiopia; The Global Fund to Fight AIDS, Tuberculosis and Malaria, Geneva, Switzerland; Federal Capital Territory Tuberculosis and Leprosy Control Programme, Abuja, Nigeria; Nigeria Tuberculosis and Leprosy Control Programme, Abuja, Nigeria; REACH Ethiopia, Hawassa, Ethiopia; UNICEF/UNDP/World Bank/WHO Special Programme for Research and Training in Tropical Diseases, World Health Organization, Geneva, Switzerland

**Keywords:** Tuberculosis, Costs, Access to healthcare, Ethiopia, Nepal, Nigeria, Yemen

## Abstract

**Background:**

A major impediment to the treatment of TB is a diagnostic process that requires multiple visits. Descriptions of patient costs associated with diagnosis use different protocols and are not comparable.

**Methods:**

We aimed to describe the direct costs incurred by adults attending TB diagnostic centres in four countries and factors associated with expenditure for diagnosis. Surveys of 2225 adults attending smear-microscopy centres in Nigeria, Nepal, Ethiopia and Yemen. Adults >18 years with cough >2 weeks were enrolled prospectively. Direct costs were quantified using structured questionnaires. Patients with costs >75^th^ quartile were considered to have high expenditure (cases) and compared with patients with costs <75^th^ quartile to identify factors associated with high expenditure.

**Results:**

The most significant expenses were due to clinic fees and transport. Most participants attended the centres with companions. High expenditure was associated with attending with company, residing in rural areas/other towns and illiteracy.

**Conclusions:**

The costs incurred by patients are substantial and share common patterns across countries. Removing user fees, transparent charging policies and reimbursing clinic expenses would reduce the poverty-inducing effects of direct diagnostic costs. In locations with limited resources, support could be prioritised for those most at risk of high expenditure; those who are illiterate, attend the service with company and rural residents.

**Electronic supplementary material:**

The online version of this article (doi:10.1186/s40249-016-0117-x) contains supplementary material, which is available to authorized users.

## Multilingual abstract

Please see Additional file 1 for translations of the abstract into the six official working languages of the United Nations.

## Background

TB is a disease of poverty mostly affecting populations with limited resources and restricted access to health services in low and middle income countries (LMICs). For this reason the WHO has included a specific financial risk protection target in the new End TB Strategy [1], that no TB affected household should experience catastrophic costs due to TB [2].

Access to services is also a major problem. Of the 9.5 million incident cases estimated by the WHO in 2013, only 6 million were reported and the rest were either not diagnosed, or diagnosed but not reported to national TB programmes (NTPs) [3]. Over 77 million smear investigations are conducted every year in the top twenty high burden countries, most of these in poor people [4]. Although drugs for first-line treatment for TB are provided for free by most NTPs [5], the financial costs incurred by the patient and their family for diagnosis remain significant [6, 7] and further costs may include hospitalisation, treatment of side effects, nutrition and others.

The onset of disease often exacerbates poverty, as the patient enters a lengthy period of expenditure on healthcare and productivity losses [8]. Direct costs start with the patient undertaking consultations to reach a diagnosis; yet these costs had been overlooked by policymakers until recently [1, 9]. Although TB diagnostics tests are free of charge in most countries, public health services often charge patients for their initial clinical consultation, non-specific medications and other diagnostic tests, such as x-rays [10, 11]. Patients must also meet the cost of displacement from home, including domestic responsibilities, subsistence and loss of earnings. Costs are augmented by the need for patients to spend several days near the health facility to complete the tests and meet health staff for clinical management decisions [12, 13].

The cost of diagnosis is not uniform for all patients and some have higher expenses than others. Identifying individuals at risk of unusually high expenditure would be of value to health programmes and policy makers to inform the development of interventions to support patients with limited resources and high expenditure. Few studies have isolated the patients’ direct and indirect costs of attending NTP health facilities for diagnosis and even fewer have compared expense patterns across countries.

This study examined the direct financial costs incurred by adults attending smear-microscopy based TB diagnostic centres in Ethiopia, Nepal, Nigeria and Yemen and the risk factors for high expenditure for diagnosis. This information was used to identify the population groups that are more likely to experience higher costs to undergo the TB diagnostic process.

## Methods

Ethical approvals for the study were obtained from the research ethics committees of the Liverpool School of Tropical Medicine, the Institutional Review Boards of the World Health Organisation and all the participating institutions in Ethiopia, Nepal, Nigeria, and Yemen. All participants gave written informed consent.

The study comprised four cross-sectional surveys using the same study protocol among patients undergoing routine smear microscopy in Ethiopia, Nepal, Nigeria and Yemen [14]. These countries were selected because the team was conducting a multi-country study to optimise the use of smear microscopy and represented high burden countries across three World Health Organization regions [14]. Adults over 18 years with chronic cough of more than 2 weeks duration attending selected health facilities were invited to participate. Participants were selected using systematic random sampling, with a maximum of 10 patients interviewed each day. In Ethiopia, participants were enrolled in Bushullo Major Health Centre, a not-for-profit mission hospital located in the outskirts of Hawassa; capital of the Southern Region; in Nepal, patients were enrolled at Tribhuvan University Teaching Hospital, a governmental referral hospital in Kathmandu; in Abuja Nigeria, patients were enrolled in Wuse District Hospital, a government general hospital and in Yemen, patients were selected from the National Tuberculosis Institute (NTI), a government referral centre and the main centre for the diagnosis of TB in Sana’a. These centres were selected as they were integral to the NTP services and large numbers of patients received a diagnosis of TB. The approximate number of patients routinely screened for TB in each centre was 260, 1000, 250 and 780 per month in Ethiopia, Nepal, Nigeria and Yemen, respectively. Individuals attending the centres self-referred or were referred by government, private or informal health care providers. A minimum of 500 patients were expected to be recruited from each study site to obtain a representative sample of patients attending the centre and to have at least 80 % power to identify risk factors with an Odds Ratio > 1.5.

Participants were interviewed using standard questionnaires to obtain demographic and clinical information, to establish whether they had travelled with company and the direct costs associated with attending the centre. The questionnaire was structured and administered face to face. Variables were defined using quantitative scales. The questionnaire had been developed over several studies preceding the study and developed jointly by all co-authors to allow for local sensitivities and pooling of expertise. Questionnaires were administered by local research staff specifically trained and employed for the study, using the local languages and took about 30 min per patient. Participants who were illiterate were supported by local staff to clarify the questions and to estimate costs. Very few patients refused to participate, and >95 % of patients consented on first approach. Patients who refused often did because they were in a hurry or (in some settings) they preferred to be interviewed with a partner who was not present at the time of the interview. A pilot study was run in all countries before the main surveys and questions were adjusted as needed. Direct costs were defined as *medical* (clinic fees or registration costs, cost of investigation or consultation, diagnostic tests and medication) and *the* costs of transportation, food and accommodation for patient and companion/s). Direct costs also included travel, overnight stays, expenditure needed to attend the second day of diagnosis and others related to the current visit. Indirect costs, defined as the loss of income for patient and household due to lost work days, incapacity to work or forced changed of occupation and substitution costs (the cost of replacing the patient in their duties) and costs for previous health service encounters were not measured.

Data were entered in a database using Epi-Info. The participants’ characteristics by country were described using summary statistics. Proportions were compared using chi square tests and means were compared using parametric and non-parametric tests for normal and skewed data, respectively. Costs were described using quartiles and expressed as the median and 25–75^th^ interquartile range (25–75 % IQR). Costs were calculated by adding the figures in the local currencies for each day of attendance and converting the day’s total to US dollars using the exchange rate at the beginning of the study. Median expenditures were described stratified by selected patient characteristics (e.g. rural/urban residency) and compared using non parametric tests. Patients with costs >75^th^ quartile were considered to have high expenditure (cases) and were compared with patients with costs <75^th^ quartile, to identify factors associated with high expenditure. Odds Ratios (OR) with 95 % confidence intervals (95%CI) were calculated and variables with p values <0.2 were entered into backward and forward logistic regressions to identify factors independently associated with high expenditure (Adjusted OR, AOR).

## Results and discussion

A total of 2225 patients were enrolled, of whom 504 were enrolled in Ethiopia, 619 in Nepal, 502 in Nigeria and 600 in Yemen. Patients had a mean (SD) age of 39 (17) years and were more likely to be male than female in all countries except Nigeria (Table 1). The majority of patients (67 %) were married or had partners; 27 % were single and few were widowed (5 %) or divorced/separated (2 %). Between 10 % (Nepal) and 47 % (Yemen) of patients resided in rural areas and the majority of urban patients resided in the town where the study health centre was located; except in Nepal, where almost half of the patients from urban areas came from other cities. The mean (SD) number of residents per household was 6.1 (4), ranging from 4.1 (3) in Nigeria to 8.6 (5) in Yemen.

**Table 1:** Patients’ demographic characteristics by country

Variables^a^		Ethiopia *N* = 504	Nepal *N* = 619	Nigeria *N* = 502	Yemen *N* = 600	All *N* = 2225
Age	(Mean, ±SD)	33.2 (14.5)	43.8 (17.8)	34.4 (11)	41.8 (18.3)	38.7 (16.6)
Gender	male: female (% male)	279: 225 (55.4)	395: 224 (63.8)	241: 258 (48.0)	329: 271 (54.8)	1244: 978 (55.9)
Marital status	Single	176 (34.9)	107 (17.3)	183 (36.5)	124 (20.7)	590 (26.5)
	With partner/married	302 (59.9)	507 (81.9)	259 (51.6)	426 (71)	1494 (67.1)
	Divorced/separated	12 (2.4)	0 (0)	13 (2.6)	10 (1.7)	35 (1.6)
	Widowed	14 (2.8)	5 (0.8)	44 (8.8)	40 (6.7)	103 (4.6)
Residence	Rural	182 (36.1)	63 (10.2)	82 (16.3)	279 (46.5)	606 (27.2)
	Same town	216 (42.9)	284 (45.9)	410 (81.7)	268 (44.7)	1178 (52.9)
	Other town	106 (21)	272 (43.9)	7 (1.4)	53 (8.8)	438 (19.7)
Literate: Illiterate (% literate)		247: 257 (49)	475: 137 (76.7)	439: 57 (87.5)	264: 336 (44.0)	1425: 787 (64)
Education	Nil	257 (51)	197 (31.8)	41 (8.2)	311 (51.8)	806 (36.2)
	Primary incomplete	117 (23.2)	37 (6)	42 (8.4)	126 (21)	322 (14.5)
	Primary complete	43 (8.5)	105 (17)	63 (12.5)	56 (9.3)	267 (12)
	Secondary	62 (12.3)	127 (20.5)	220 (43.8)	61 (10.2)	470 (21.1)
	Tertiary	24 (4.8)	153 (24.7)	133 (26.5)	46 (7.7)	356 (16)
	Missing	1 (0.2)	0 (0)	3 (0.6)	0 (0)	4 (0.2)
Working: not working (% working)		235: 269 (46.6)	202: 417 (32.6)	368: 131 (73.3)	164: 436 (27.3)	969: 1253 (43.6)
Mean (SD) residents in household		5.7 (2.9)	5.5 (3)	4.1 (3)	8.6 (5)	6.1 (4)

^a^Data represent frequency (%), unless otherwise specified. Sex, residency, marital and work status were missing for 3 patients and literacy for 13 patients

The majority of patients were accompanied by another person in all countries except Nigeria, as shown in Table 2. The accompanying person was a relative other than the spouse in 77 % of patients, the spouse in 15 % and 8 % were accompanied by other people. Few patients walked to the clinics and the most common transport methods were buses in Nigeria, Nepal and Ethiopia and cars or taxis in Yemen. Horse cart and motorbike travel were also common in Ethiopia (26 %) and Nepal (17 %), respectively. The median (25–75 % IQR) travel time to the health centre was 40 (20–66) minutes. Travel time was shortest in Nepal (25; 15–30 min) and longest in Yemen (60, 30–150 min). The majority of patients intended to spend the night at home the first day of consultation (66 %), except in Nepal where 46 % planned to stay with a relative. Very few patients paid for hotel accommodation. Transport patterns for the second day of consultation where similar, except in Yemen where patients realised that there was a low cost taxi service and a lower number decided to travel by private car the second day (data not shown).

**Table 2:** Characteristics of patients attending the clinics

Variables^a^		Ethiopia *N* = 504	Nepal *N* = 619	Nigeria *N* = 502	Yemen *N* = 600	All *N* = 2225
Alone: With company (% accompanied)		129:373 (74)	181:435 (70.3)	320:177 (35.3)	91:509 (84.8)	721:1494 (67.1)
Accompanying person	Spouse	61 (16.4)	62 (14.3)	50 (28.2)	51 (10)	224 (15)
	Other relative	276 (74)	336 (77.2)	94 (53.1)	439 (86.2)	1145 (76.6)
	Friend	13 (3.5)	17 (3.9)	19 (10.7)	17 (3.3)	66 (4.4)
	Neighbour	7 (1.9)	10 (2.3)	1 (0.6)	0 (0)	18 (1.2)
	Other	16 (4.3)	9 (2.1)	13 (7.3)	2 (0.4)	40 (2.7)
Travel time, median (25–75 IQR), min		45 (27–120)	25 (15–30)	45 (30–60)	60 (30–150)	40 (20–66)
Transport day 1	Walking	67 (13.3)	92 (14.9)	12 (2.4)	12 (2)	183 (8.2)
	Carried	6 (1.2)	0 (0)	2 (0.4)	0 (0)	8 (0.4)
	Bicycle	14 (2.8)	7 (1.1)	0 (0)	0 (0)	21 (0.9)
	Cart/horse	129 (25.6)	5 (0.8)	0 (0)	4 (0.7)	138 (6.2)
	Motorbike	1 (0.2)	104 (16.8)	2 (0.4)	3 (0.5)	110 (4.9)
	Bus	148 (29.4)	303 (48.9)	303 (60.4)	127 (21.2)	881 (39.6)
	Car	33 (6.5)	14 (2.3)	76 (15.1)	203 (33.8)	326 (14.7)
	Taxi	103 (20.4)	83 (13.4)	101 (20.1)	250 (41.7)	537 (24.1)
	Other	1 (0.2)	8 (1.3)	1 (0.2)	1 (0.2)	11 (0.5)
Transport day 2	Walking	118 (23.4)	104 (16.8)	13 (2.6)	12 (2)	247 (11.1)
	Carried	3 (0.6)	2 (0.3)	2 (0.4)	1 (0.2)	8 (0.4)
	Bicycle	13 (2.6)	9 (1.5)	0 (0)	0 (0)	22 (1)
	Cart/horse	181 (35.9)	5 (0.8)	0 (0)	5 (0.8)	191 (8.6)
	Motorbike	1 (0.2)	104 (16.8)	1 (0.2)	3 (0.5)	109 (4.9)
	Bus	44 (8.7)	298 (48.1)	309 (61.6)	143 (23.8)	794 (35.7)
	Taxi	108 (21.4)	72 (11.6)	73 (14.5)	327 (54.5)	580 (26.1)
	Other	5 (1)	9 (1.5)	38 (7.6)	0 (0)	52 (2.3)
Accommodation	Home	358 (71)	267 (43.1)	437 (87.1)	407 (67.8)	1469 (66)
	Relative	60 (11.9)	282 (45.6)	5 (1)	123 (20.5)	470 (21.1)
	Hotel	66 (13.1)	40 (6.5)	0 (0)	68 (11.3)	174 (7.8)
	Street	2 (0.4)	0 (0)	1 (0.2)	0 (0)	3 (0.1)
	Shift	1 (0.2)	6 (1.0)	0 (0)	0 (0)	7 (0.3)
	Hospital	15 (3.0)	20 (3.2)	54 (10.8)	2 (0.3)	91 (4.1)

^a^Data are frequency (%), unless specified. Patients’ data missing for whether they attended with company (i10,) person accompanying (1), accommodation (11 and transport (18). Min = minutes. P values for all comparisons between countries < 0.05, except for accompanying person being the spouse (*p* = 0.26) and Being carried (*p* = 0.69)

### Costs of attending the clinic

Costs associated with the two-day clinic attendance are summarised in Table 3. Second day expenses were similar to first day expenses. The median costs were higher in Yemen ($11.89) and Nepal ($8.22) and lower in Nigeria ($5) and Ethiopia ($1.47). The most significant expenses were due to the clinic costs (except Nigeria) and transport. Miscellaneous expenses were frequently reported in Nepal (83 % of patients) and were rare in Yemen (12 %), Nigeria (3 %) and Ethiopia (0 %). Although rare in Yemen, these expenses were significant. Very few patients paid for overnight accommodation in all countries and the median (25 % & 75 % IQR) expenditure was 0 in all countries.

**Table 3:** Median and IQR costs incurred for attending the clinic

Variable^a^	Ethiopia ETB *N* = 504	Nepal NPR *N* = 619	Nigeria NGN *N* = 502	Yemen YER *N* = 600
Proportion reporting miscellaneous expenses, N (%)^b^	0 (0)	509 (82.6 %)	13 (2.6 %)	73 (12.2 %)
Clinic costs	8 (5; 8)	300 (300; 300)	0 (0; 150)	450 (250; 450)
Transport	16 (4; 36)	48 (0; 100)	400 (200; 600)	2000 (800; 3400)
Food	0 (0; 12)	0 (0; 0)	200 (0; 400)	0 (0; 400)
Overnight accommodation	0 (0; 0)	0 (0; 0)	0 (0; 0)	0 (0; 0)
Miscellaneous expenditure	0 (0; 0)	300 (200; 300)	500 (200; 1000)	1000 (500; 2000)
Total	ETB 24 (11; 58)	NPR 604 (500; 900)	NGN 760 (420; 1400)	YER 2550 (1400; 4850)
Total (US $)	$1.47	$8.22	$5.00	$11.89
Proportion of poor/general population (H) using MPI^c^	0.900	0.647	0.635	0.525
Proportion of poor/general population < $1.25/day^d^	0.390	0.551	0.644	0.175
Conversion rate to USD	1 = 0.061	1 = 0.013	1 = 0.007	1 = 0.005

^a^Values are medians and 25–75 % interquartile ranges (IQR) and given in the local currency, unless specified otherwise

^b^Data missing for 10 patients (2 in Ethiopia, 3 in Nepal and 5 in Nigeria)

^c^Figures derived from the Multidimensional Poverty Index [34]

^d^Proportion of people living on < $1.25/day, World Development Indicators, World Bank 2009

The costs of attending the clinic stratified by the patients’ characteristics are described in Table 4. Older patients, those attending the clinic with company, residing in rural areas or coming from other towns and patients not working, had higher median costs than younger patients, those attending alone, residing in the same town or working. Farmers, students and housewives had higher median costs than patients with other occupations. Patients with a confirmed TB diagnosis (smear and culture positive) had higher costs than patients without laboratory confirmation and expenses were similar among males and females.

**Table 4:** Median costs incurred according to patients’ characteristics

	Variable^a^	Ethiopia *N* = 504		Nepal *N* = 619		Nigeria *N* = 502		Yemen *N* = 600	
		N	ETB	N	NPR	N	NGN	N	YER
Age (years)	<20	52	21 (8; 48)	37	600 (500; 740)***	13	1100 (850; 2300)****	54	2240 (1400; 3850)
	20–49	367	24 (11; 58)	332	600 (500; 858)	444	725 (400; 1390)	312	2550 (1400; 4795)
	≥50	83	26 (12; 60)	250	640 (560; 980)	40	800 (525; 1800)	232	2760 (1445; 5050)
Gender	Male	278	24 (11; 56)	395	610 (500; 976)	241	700 (420; 1400)	328	2525 (1305; 4650)****
	Female	224	24 (11: 60)	224	600 (500; 849)	256	800 (480; 1400)	271	2600 (1600; 5450)
Residency	Rural	182	43 (24; 79)*	63	725 (460; 1890)*	82	700 (480; 1400)	279	3890 (2250; 6250)*
	Same town	215	12 (5; 24)	284	598 (500; 749)	408	760 (400; 1400)	267	1650 (890; 2654)
	Other town	105	56 (40; 82)	272	648 (572; 895)	7	1660 (850; 1800)	53	4250 (2450; 7360)
Companions	Alone	129	9 (5; 14)*	181	600 (500; 750)**	320	645 (400; 1100)*	90	1420 (800; 2450)*
	Accompanied	373	38 (24; 72)	435	620 (500; 980)	177	1100 (600; 1900)	509	2850 (1650; 5250)
Diagnosis	Completed	488	24 (11; 58)	602	600 (500; 885)	423	750 (400; 1400)	569	2550 (1400; 4890)
			$1.47		$8.16		$4.94		$11.89
	Incomplete	14	27.5 (8; 48)	16	868 (400; 1188)	47	750 (500; 1400)	30	2675 (1650; 4050)
			$1.68		$11.81		$4.94		$12.47
Smear result	Positive	111	28 (13; 53)	71	700 (560; 1160)***	85	800 (480; 1400)	120	2610 (1450; 4850)
	Negative	389	24 (11; 58)	545	600 (500; 850)	403	740 (400; 1400)	476	2550 (1400; 4930)
Culture	Positive	141	29 (13; 56)****	73	672 (580; 1160)***	80	770 (400; 1400)	150	2535 (1400; 4800)
	Negative	345	24 (11; 58)	515	600 (500; 840)	399	760 (420; 1400)	430	2555 (1450; 5050)
Illness duration (weeks)	1–2	90	24 (11; 48)****	63	540 (270; 1200)**	133	750 (420; 1400)	162	2625 (1510; 5050)
	3–4	153	24 (9; 52)	153	596 (500; 780)	144	800 (400; 1400)	140	2400 (1325; 4610)
	5–11	120	38 (17; 64)	129	600 (540; 950)	91	800 (400; 1440)	131	2850 (1350; 4890)
	≥12	137	24 (11; 72)	271	640 (560; 976)	118	700 (500; 1220)	153	2480 (1550; 4750)
Cough duration (weeks)	1–2	95	24 (11; 48)****	80	565 (300; 1148)**	165	800 (400; 1500)	196	2650 (1455; 5100)
	3–4	160	24 (9; 50)	169	600 (500; 800)	134	740 (400; 1380)	132	2450 (1450; 4725)
	5–11	115	36 (16; 64)	130	630 (540; 1020)	84	800 (490; 1440)	118	2425 (1250; 4750)
	≥12	128	24 (11.5; 72)	238	623 (580; 900)	104	700 (500; 1370)	138	2515 (1610; 4750)
Employment status	Working	234	24 (10; 56)	202	598 (500; 780)**	367	700 (400; 1300)**	163	2150 (1090; 4050)**
	Not working	268	28 (11.5; 63)	417	640 (540; 900)	130	1000 (520; 1600)	436	2650 (1580; 5100)
Occupation	Farmer	125	52 (24; 80)*	133	648 (552; 980)	32	800 (535; 1600)***	140	3375 (2000; 6050)*
	Student	47	24 (11; 52)	66	640 (500; 900)	46	1150 (550; 1900)	58	2250 (1250; 4050)
	Housewife	121	42 (24; 73)	146	611.5 (500; 848)	22	690 (400; 1040)	210	2850 (1650; 6050)
	Labourer	38	11 (5; 21)	21	600 (480; 1080)	21	1200 (640; 1440)	71	2050 (1120; 3250)
	Merchant	32	22.5 (8; 52)	97	600 (536; 720)	1	20000 (−; −)	8	1925 (975; 2950)
	Government	45	13 (7; 27)	33	600 (540; 700)	86	720 (400; 1300)	61	2500 (1250; 4250)
	Other	92	16 (8; 27.5)	117	600 (500; 975)	289	700 (400; 1320)	51	1300 (800; 2950)

^a^Values are medians and 25–75 % interquartile ranges (IQR) and given in the local currency, unless specified otherwise

*P <0.001, **P < 0.01, ***P < 0.05, ****P < 0.2

A total of 538 cases with expenditure >75^th^ centile (high expenditure) and 1679 patients with expenditure <75^th^ centile (controls) were analysed. Factors associated with high patient expenditure at the univariate and multivariate analysis are shown in Table 5.

**Table 5:** Risk factors for high patient expenditure, odds ratios (OR), adjusted odds ratios (AOR) with 95 % Confidence intervals (95 % CI) by country

	Ethiopia		Nepal		Nigeria		Yemen	
Variable	OR	AOR	OR	AOR	OR	AOR	OR	AOR
Age ≥50	1.1 (0.6–1.8)		1.3 (0.9–1.8)		1.6 (0.8–3.2)		1.3 (0.9–1.9)	
Male	0.9 (0.6–1.4)		1.4 (0.9–2.1)	2.0 (1.3–3.2)	1.1 (0.7–1.6)		0.7 (0.5–1.1)	
Married or with partner	1.9 (1.3–3.0)		0.8 (0.5–1.3)		1.0 (0.7–1.5)		1.1 (0.7–1.7)	
Rural/other town residency	23.6 (10.2–55)	15.3 (6.5–36.0)	1.5 (1.0–2.2)		1.1 (0.6–1.8)		6.9 (4.2–11.3)	6.7 (4.1–11.0)
Illiterate	1.9 (1.3–2.9)		1.7 (1.1–2.6)	2.5 (1.5–4.0)	2.4 (1.4–4.3)	1.9 (1.0–3.4)	1.7 (1.2–2.5)	
Education nil or primary	2.5 (1.3–4.8)		1.4 (1.0–2.1)		1.1 (0.7–1.7)		1.9 (1.1–3.3)	
Accompanied	19.7 (6.1, 63)	7.5 (2.3–25.2)	1.5 (1.0–2.2)		2.7 (1.8–4.2)	2.5 (1.6–3.9)	3.4 (1.7–7.0)	3.1 (1.5–6.5)
Smear-positive	0.9 (0.5–1.4)		2.2 (1.3–3.7)		0.8 (0.5–1.5)		1.0 (0.6–1.5)	
Culture-positive	1.0 (0.6–1.6)		2.2 (1.3–3.7)	2.3 (1.3–3.8)	1.0 (0.6–1.7)		0.9 (0.6–1.4)	
Illness ≥ 12 weeks	1.6 (1.0–2.4)		1.2 (0.8–1.7)		0.9 (0.5–1.5)		0.9 (0.6–1.5)	
Cough ≥ 5 to 11 weeks	1.3 (0.8–2.0)		1.3 (0.9–2.1)		1.2 (0.7–2.0)		1.0 (0.6–1.6)	
Not working	1.2 (0.8–1.9)		1.1 (0.8–1.7)		1.7 (1.1–2.7)		1.4 (0.9–2.1)	
Farmer, student, housewife, labourer	3.8 (2.2–6.5)		1.1 (0.8–1.6)		1.4 (0.9–2.3)		2.3 (1.3–4.0)	

Although there were considerable differences between countries, residing in a rural area/other town (OR (95 % CI) 23.6 (10.2–55); 1.5 (1.0–2.2) and 6.9 (4.2–11.3), respectively), having low education (2.5 (1.3–4.8), 1.4 (1.0–2.1) and 1.9 (1.1–3.3), respectively) and attending with company (19.7 (6.1, 63), 1.5 (1.0–2.2) and 3.4 (1.7–7.0), respectively) were risk factors for high expenditure in Ethiopia, Nepal and Yemen at the univariate level. Of these, illiteracy (OR (95 % CI) 2.4 (1.4–4.3) and being accompanied (OR (95 % CI) 2.7 (1.8–4.2) were also statistically significant in Nigeria. Variables independently associated with high expenditure in the multivariate analysis, were attending the clinic with company (Ethiopia (AOR 7.5 (2.3–25.2), Yemen (AOR 3.1 (1.5–6.5) and Nigeria (AOR 2.5 (1.6–3.9)), residing in rural areas and other towns (Ethiopia (AOR 15.3 (6.5–36.0) and Yemen 6.7 (4.1–11.0)), illiteracy (Nigeria (AOR 1.9 (1.0–3.4) and Nepal (AOR 2.5 (1.5–4.0)) and males with bacteriologically confirmed TB (Nepal, AOR 2.0 (1.3–3.2)).

Many illnesses are associated with poverty and individuals often consider their financial position before attending health facilities. TB is no exception. As a disease of poverty, TB is associated with many patients never attending health services, attending late, or dropping out after initiation of the diagnostic process [10, 15, 16]. As TB diagnostic services are not available in all health facilities, patients often express concerns about multiple consultations, service fees, travel expenses and lost time and opportunity costs [17]. This analysis confirms that the direct costs sustained by patients undergoing a diagnosis of TB across multiple settings are substantial. A large component of these are associated with clinic costs, transport and patients attending the services with company. According to the Multidimensional Poverty Index, of the four countries Yemen, ranked as the least poor at the time of the study, followed by Nigeria, Nepal and Ethiopia [2]. Although it is difficult to compare costs and expenditure directly between countries, as living costs and income were very different across study settings, our findings identified remarkable similarities.

Clinic user fees comprised a common and significant cost. These fees are known to have a negative impact on general health service utilisation and this is likely to be more prominent in TB patients with limited financial resilience [18]. Clinic costs for attending TB clinics in the study comprised consultation fees, smear microscopy, X-rays and blood tests to screen for other diseases [19]. Furthermore, although not captured in the study, bacteriologically negative cases may undergo further consultation and testing, pay for further visits and have higher expenditure than smear-positive cases.

Although some NTPs retrospectively reimburse diagnostic costs to patients with TB, only 10–20 % of patients receive a TB diagnosis. Patients therefore need to be prepared to meet expenses up front; in practise 80–90 % will not be reimbursed and these fees are likely to be a major deterrent for attending diagnostic services. Furthermore, we have documented elsewhere that patients are often overcharged or pay under-the-counter fees to speed up test results or to be seen earlier than others [20]. These expenses are not documented in their receipts and where reimbursement does occur, patients are often only partially reimbursed.

Transport costs also contribute significantly to expenditure in Ethiopia, Nigeria and Yemen, where a high number of patients travel from other towns and rural areas and use buses and taxis. In Ethiopia, the costs reflect the predominantly rural population of the country and the cost of transport from areas with limited road and public transport infrastructure. In Yemen, collective public transport is limited to the main towns and women often rely on private vehicles to attend the fairly centralised diagnostic services. In Nigeria, buses were very limited within the metropolitan areas of the Federal Capital at the time of the study and people relied on share minibuses or taxis to move from the slums surrounding the metropolitan areas. Although transport costs were incurred for different reasons in each location, they represented more than half of patient expenditure to attend diagnostic centres and are thus a major barrier. Addressing these issues would require long term infrastructural development. However interim solutions such as recruiting community volunteers or community workers to organise transport and the provision of support funds that facilitate transport could be explored [21]. Similar issues occur for other diseases such as HIV/AIDS, the management of accidents and emergencies and complicated deliveries.

A high proportion of patients attended with company in all study settings. This was especially prominent in Yemen, where 4 out of 5 patients came accompanied, and significantly increased the costs associated with displacement. In Yemen, gender norms meant that women in particular were required to be accompanied by a male relative and faced particular challenges in accessing a TB diagnosis [20]. The need for company also reflects cultural practices underpinning support to a person perceived to have a mortal illness - in Ethiopia, for example, TB is equated with lung cancer - and the frailty of patients with chronic and debilitating conditions. Rural residents were more likely to attend with company in all countries, which is probably the reason why these patients had higher expenditure in Ethiopia and Yemen than patients from urban areas [11].

Our findings indicate that despite the differences in the settings, patients across LMICs experience many similarities in the type of costs associated with clinic attendance and that it might be feasible to identify patients at risk of high expenditure by conducting a simple questionnaire when they present to diagnostic centres. This is particularly relevant in the context of the Global Plan to Stop TB 2016–2020, which aims to eliminate the number of families facing catastrophic costs due to TB [1, 22]. In the study context, additional support made available by NTPs could be channelled to those most at risk of high costs, including illiterate patients, those originating from rural areas or attending with company. This approach could also reimburse expenses to *all* patients investigated for TB; independently of whether the diagnosis is confirmed.

This analysis, however, has several limitations, beginning with the sampling strategy, which carried a risk of selection bias. Participants were recruited using systematic random sampling, rather than randomly, as this suited the objectives of the larger clinical trial [14]. Patients arriving at the beginning of the day might have had different characteristics from those arriving later. We can hypothesise that the former might have resided more locally, or conversely, have travelled the previous day from afar and stayed overnight. Patients arriving early might have been better prepared. Next, second day costs were calculated according to patients’ predicted expenses, rather than the actual costs incurred. Moreover, all costs were self-reported, rather than observed by investigators. Actual and predicted expenditure might have been expressed differently by different subgroups of the population and different cultures, as mediated by established social hierarchies, gender roles, economic standing and the distribution of power, to name but a few modifiers. For example, costs which might be overstated by patients in one setting in the hope of financial remuneration might be underreported in another out of individual pride. Women who do not have access to household finances might also be unaware of the full cost of attendance. Calculation of costs as a proportion of individual income would have provided a more complete picture of the economic burden for the individual and their family, however including the many variables involved in these calculations was not possible within the confines of a short addition to a large survey and asking participants to disclose their income was considered unreliable.

The costs described here represent only a proportion of the economic barriers faced by individuals with symptoms of TB, as the cost of previous healthcare, visits to the private sector and indirect costs - such as loss of employment and work time – which may constitute a major part of patients’ outgoings, were not documented [19, 20, 23–26]. Opportunity costs tend to be higher for people living in poverty, who for the most part work in the informal sector and are vulnerable to loss of income or dismissal from work [27]. In countries with high HIV prevalence, this vulnerability is heightened, as the population perceives that patients with TB are likely to be co-infected with HIV [28]. Furthermore, a large proportion of symptomatic adults do not attend diagnostic centres and these individuals often have fewer financial and social resources at their disposal [20]. Financial barriers could be alleviated at the point of care through further decentralisation of health services [29], the provision of free diagnostic services, transport for remote populations and/or mobilisation of services to remote communities [28, 30, 31].

## Conclusion

The costs incurred by patients are substantial and share common patterns across countries. Removing user fees, transparent charging policies and reimbursing clinic expenses would reduce the poverty-inducing effects of direct diagnostic costs. In locations with limited resources, support could be prioritised for those most at risk of high expenditure; those who are illiterate, attend the service with company and rural residents.

Poverty constitutes a major access barrier for symptomatic adults in low income countries, as recently recognised by the Sustainable Development Goals (SMGs) [32]. Poverty is often compounded by low education and health information, leading to misconceptions of the disease and disempowerment. Rural and urban residence also determines access to diagnostic services. Addressing poverty is likely to be the most crucial factor in determining the success or failure of the Global Health Plan to Stop TB 2016–2020 as a significant public health problem [1] and recognised as major impediment for the achievement of the TB component of the SMGs [17, 33].

### Ethics statement

The protocol (International Standard Randomized Controlled Trial Number Register ISRCTN53339491) was approved by the WHO Ethics Review Committee, the Liverpool School of Tropical Medicine Ethics Research Committee, and the national and institutional ethics committees of the four countries. Consent and information sheets were translated, and informed witnessed written or oral consent was obtained.

## Additional file

Additional file 1: Multilingual abstracts in the six official working languages of the United Nations. (PDF 314 kb)

## Abbreviations

AOR: adjusted odds rations
CI: Confidence Intervals
DFiD: Department for International Development
ESRC: Economic and Social Research Council
IQR: interquartile range
NTI: National Tuberculosis Institute
NTPs: national TB programmes
OR: Odds Ratios
SD: standard deviation
TB: tuberculosis
SDGs: Sustainable Development Goals
