# Supplementary material for: Patients direct costs to undergo TB diagnosis
Source: Infect Dis Poverty. 2016 Mar 24;5:24. doi: 10.1186/s40249-016-0117-x (PMC4806474; doi:10.1186/s40249-016-0117-x)
Supplement: Additional file 1: — Multilingual abstracts in the six official working languages of the United Nations. (PDF 314 kb) [file 40249_2016_117_MOESM1_ESM.pdf]

Translation of the abstract into the five official working languages of the United Nations

**التكاليف المباشرة على المرضى الذين يخضعون لتشخيص أعراض مرض السل**

راشيل أندرسون دو كويفاس، لوفيت لوسون، نجلاء السنبلي، ناشر الأغبري، إيزابيل عربيد، جيفان شيرتشاند، امينيونو نامدي، أبراهام أسيفا، محمد ياسين، صديق عبد الرحمن، جوشوا أوباسانيا، أولانريواجو اولاديميجي، دانييل جيميتشو، سالي ثيوبولد، أندرو رامزي، برتيل سكواير، لويس كويفاس

**ملخص**

**نبذة:** يعتبر العائق الرئيسي في علاج السل هو عملية تشخيص الأعراض التي تتطلب القيام بزيارات متعددة؛ واستخدام نظم مختلفة في توصيف التكاليف المرتبطة بالتشخيص، كما أنها غير قابلة للمقارنة مع غيرها من الأنظمة.

**الأساليب:** تركز هدفنا على وصف التكاليف المباشرة التي يتكبدها الكبار الذين يحضرون مراكز تشخيص أعراض السل في البلدان الأربعة والعوامل المرتبطة بمصروفات إجراءات التشخيص. شملت الدراسة أربع دراسات استقصائية مقطعية بين المرضى الذين يخضعون للفحص الروتيني المجهري للعينات في إثيوبيا، ونيبال، ونيجيريا، واليمن. الكبار > 18 سنة الذين لديهم أعراض سعال > 2 أسبوع تم تسجيلهم مقدماً. تم احتساب التكاليف المباشرة كمياً باستخدام استبيانات هيكلية. المرضى الذين يعانون من التكاليف > 75 ربعياً اعتبرت تواجه ارتفاع النفقات (حالات) وقورنت مع المرضى الذين يعانون من التكاليف < 75 ربعياً لتحديد العوامل المرتبطة بارتفاع النفقات.

**النتائج:** ظهر أن السبب في النفقات الرئيسية يعود إلى رسوم العيادة والانتقال. وحضر معظم المشاركين إلى المراكز مع رفاقهم. كما ارتبط ارتفاع النفقات بحضور المريض مع مرافقين الذين يقيمون في المناطق الريفية/ بلدات أخرى وكذلك بسبب الأمية.

**الاستنتاجات:** التكاليف التي يتكبدها المرضى تعتبر هائلة وتشترك مع خصائص مشتركة مع بلدان أخرى. ومن خلال استبعاد رسوم الاستخدام واتباع سياسات أكثر شفافية في فرض الرسوم والتعويض عن المصروفات المدفوعة إلى العيادات، فإنه يمكن الحد من أثار التكاليف المباشرة للتشخيص على زيادة حدة الفقر. وفي الأماكن ذات الموارد المحدودة، يمكن ترتيب أولويات الدعم لهؤلاء الأكثر تعرضا لخطر ارتفاع النفقات؛ وأولئك الذين يعانون من الأمية، الذين يحضرون مع مرافقين لهم وكذلك القادمين من المناطق الريفية.

Translated from English version into Arabic by Lamya, through

TRANSLATORS
WITHOUT BORDERS

**结核病人诊断的直接成本分析**

Rachel M. Anderson de Cuevas, Lovett Lawson, Najla Al-Sonboli, Nasher Al-Aghbari, Isabel Arbide, Jeevan B. Sherchand, Emenyonu E Nnamdi, Abraham Aseffa, Mohammed A. Yassin, Saddiq T. Abdurrahman, Joshua Obasanya, Oladimeji Olanrewaju, Daniel Gemechu, Sally J. Theobald, Andrew Ramsay, S. Bertel Squire, Luis E. Cuevas

**摘要**

**引言：**治疗结核病的一个主要障碍就是需要多次就诊。不同方法进行诊断，其相关支出的描述也不同，且不具有可比性。

**方法：**在 4 个国家对来结核病诊断中心就诊的成人患者的诊断直接成本以及与诊断支付相关的因素进行分析。本研究包括在埃塞俄比亚、尼泊尔、尼日利亚和也门患者中进行的常规涂片镜检的 4 个横断面调查。将年龄大于 18 周岁，咳嗽时间长于两周的成年人纳入研究。采用结构式问卷调查对直接成本进行量化。患者支出大于第三四分位数(75%)被认为花费较大，并与支出小于第三四分位数(75%)患者相比来确定高支出

的相关因素。
**结果：**研究结果表明，最主要的支出为门诊费用和交通费用。大部分受试者到诊断中心就诊均有人员陪同，高支出与陪同人员的参与，居住在农村地区/其他城镇及文盲有关。
**结论：**研究结果表明患者花费是很大的，且在各国之间有共同的模式。透明的收费政策和门诊费用的报销将有助于降低直接诊断成本造成的致贫效应。在资源有限的地区，建议优先支助有较高支付危险的人群，如文盲、有人员陪同的就诊者以及农村居民。

Translated from English version into Chinese by Feng Xinyu, edited by Yang Pin, through

**Coûts directs assumés par des patients dans le cadre du diagnostic de la TB**

Rachel M. Anderson de Cuevas, Lovett Lawson, Najla Al-Sonboli, Nasher Al-Aghbari, Isabel Arbide, Jeevan B. Sherchand, Emenyonu E Nnamdi, Abraham Aseffa, Mohammed A. Yassin, Saddiq T. Abdurrahman, Joshua Obasanya, Oladimeji Olanrewaju, Daniel Gemechu, Sally J. Theobald, Andrew Ramsay, S. Bertel Squire, Luis E. Cuevas

**Résumé**
**Contexte :** Un processus de diagnostic requérant plusieurs visites constitue un important obstacle au traitement de la TB. Les descriptions des coûts supportés par les patients associés au diagnostic reposent sur différents protocoles et ne sont pas comparables.
**Méthodes :** Notre objectif a été de décrire les coûts directs supportés par des adultes se rendant auprès de centres de diagnostic de la TB dans quatre pays différents ainsi que les facteurs associés aux dépenses de diagnostic. L'étude s'est composée de quatre sondages transversaux parmi des patients subissant des examens microscopiques de frottis de routine en Éthiopie, au Népal, au Nigeria et au Yémen. Des adultes > 18 ans atteints de toux > 2 semaines ont été recrutés de manière prospective. Les coûts directs ont été quantifiés en utilisant des questionnaires structurés. Nous avons considéré que les patients dont les coûts > 75ème percentile supportaient des dépenses élevées (cas) et nous les avons comparé avec des patients dont les coûts < 75ème percentile afin d'identifier des facteurs associés à des dépenses élevées.
**Résultats :** Les dépenses les plus significatives se rapportaient aux frais hospitaliers et aux frais de transport. La plupart des participants se sont présentés aux centres en étant accompagnés. Des dépenses élevées étaient associées au fait de se présenter en étant accompagné(e), à la résidence dans des régions rurales/d'autres villes et à l'analphabétisme.
**Conclusions :** Les coûts supportés par les patients sont substantiels et présentent des modèles communs dans les différents pays. La suppression des frais supportés par les patients, l'élaboration de politiques tarifaires transparentes et le remboursement des frais hospitaliers permettraient une réduction des effets des frais de diagnostic directs engendrant une certaine pauvreté. Dans les régions aux ressources limitées, un soutien pourrait être accordé en priorité aux personnes les plus à risque de présenter d'importantes dépenses, aux personnes analphabètes, aux personnes qui se rendent au service en étant accompagnées ainsi qu'aux personnes résidant dans des zones rurales.

Translated from English version into French by eric ragu, through

## Прямые расходы пациентов на прохождение диагностики туберкулеза

Рейчел М. Андерсон де Куэвас, Ловетт Лосон, Наджла Аль-Сонболи, Нашер Аль-Агбари, Изабель Арбиде, Дживан б. Шерчанд, Эменьону Э Ннамди, Абрахам Асеффа, Мохаммед А. Яссин, Саддик Т. Абдуррахман, Джошуа Обасанья, Оладимеджи Оландеваджу, Даниэль Гемечу, Салли Дж. Теобальд, Эндрю Расей, С. Бертел Сквайр, Луис Э. Куэвас

**Аннотация**

**Предпосылки:** Основным препятствием для лечения туберкулеза является то, что диагностический процесс требует многократных явок. Описания расходов пациента, связанных с диагностикой, производятся по различным протоколам и являются несовместимыми.

**Методы:** Мы намеревались описать прямые расходы, которые несут взрослые, посещающие центры диагностики туберкулеза в четырех странах и факторы, связанные с расходами на диагностику. Исследование состояло из четырех перекрестных опросов пациентов, проходящих рутинную микроскопию мазка в Эфиопии, Непале, Нигерии и Йемене. Данные взрослых старше 18-ти лет с кашлем дольше двух недель регистрировались во временной перспективе. Прямые затраты количественно оценивались с помощью опросников. При идентификации факторов, связанных с высокими расходами, пациенты с расходами в квартили больше 75% считались представляющими (заболевание) с высокими расходами и сравнивались с пациентами с расходами в квартили меньше 75%.

**Результаты:** Наиболее значительные расходы приходились на выплаты клиникам и транспорт. Большинство участников посещало центры с сопровождающими. Высокие расходы были связаны с посещением с сопровождающими, проживанием в сельской местности/другом городе и неграмотностью.

**Выводы:** Расходы, которые несут пациенты, являются значительными и имеют общий характер в разных странах. Снятие оплаты с пользователя, прозрачная политика взимания оплаты и возмещение расходов клиник должны снизить усиливающие бедность эффекты стоимости прямой диагностики. В местах с ограниченными ресурсами следовало бы сделать приоритетной помощь тем, кто наиболее подвержен риску высоких расходов: неграмотным, посещающим службу в сопровождении других и сельским жителям.

Translated from English version into Russian by Alexander Somin, through

## Costos directos del paciente para recibir diagnóstico de TB

Rachel M. Anderson de Cuevas, Lovett Lawson, Najla Al-Sonboli, Nasher Al-Aghbari, Isabel Arbide, Jeevan B. Sherchand, Emenyonu E Nnamdi, Abraham Aseffa, Mohammed A. Yassin, Saddiq T. Abdurrahman, Joshua Obasanya, Oladimeji Olanrewaju, Daniel Gemechu, Sally J. Theobald, Andrew Ramsay, S. Bertel Squire, Luis E. Cuevas

**Resumen**

**Antecedentes:** Un importante impedimento para el proceso de diagnóstico de la TB es que requiere de múltiples consultas. Las descripciones de los costos para el paciente asociados al diagnóstico utilizan distintos protocolos y no pueden compararse.

**Métodos:** Nuestro objetivo fue describir los costos directos en los que incurren pacientes adultos que asisten a centros de diagnóstico para la TB en cuatro países y los factores asociados a los gastos para el diagnóstico. El estudio estuvo compuesto de cuatro encuestas transversales a pacientes a los que se les practicaba microscopía electrónica de barrido de rutina en Etiopía, Nepal, Nigeria y Yemen. Se inscribió en el estudio a adultos mayores de 18 años con tos por más de dos semanas. Se cuantificaron los costos directos mediante el uso de cuestionarios estructurados. Se consideró que aquellos pacientes que tenían costos mayores al cuartil 75 tenían gastos elevados (casos) y se los comparó con pacientes que tenían costos menores al cuartil 75 para identificar los factores asociados a dichos gastos elevados.

**Resultados:** Los gastos más significativos fueron debido a aranceles de la clínica y transporte. La mayoría de los participantes asistieron a los centros con acompañantes. Los gastos elevados estaban asociados al asistir con acompañante, vivir en áreas rurales u otras aldeas y a analfabetismo.

**Conclusiones:** Los costos en los que incurren los pacientes son importantes y tienen patrones en común en los distintos países. El quitar los aranceles de usuario, la implementación de políticas de cobro transparentes y el reembolso de los gastos de la clínica reducirían los efectos inductores de pobreza de los costos directos de diagnóstico. En localidades con recursos limitados, debería darse prioridad al apoyo para aquellas personas que corren riesgo de mayores gastos; los analfabetos, a quienes asisten al servicio con acompañantes y a los residentes rurales.

Translated from English version into Spanish by Mpgorgone, through

TRANSLATORS
WITHOUT BORDERS
